# Wearable Activity Tracker Use Among Australian Adolescents: Usability and Acceptability Study

**DOI:** 10.2196/mhealth.9199

**Published:** 2018-04-11

**Authors:** Nicola D Ridgers, Anna Timperio, Helen Brown, Kylie Ball, Susie Macfarlane, Samuel K Lai, Kara Richards, Kelly A Mackintosh, Melitta A McNarry, Megan Foster, Jo Salmon

**Affiliations:** ^1^ Institute for Physical Activity and Nutrition School of Exercise and Nutrition Sciences Deakin University Burwood Australia; ^2^ Jean Hailes for Women’s Health Organisation Melbourne Australia; ^3^ Learning Futures Deakin University Burwood Australia; ^4^ Applied Sports, Technology Exercise and Medicine Research Centre College of Engineering Swansea University Swansea United Kingdom

**Keywords:** qualitative research, fitness trackers, physical activity

## Abstract

**Background:**

Wearable activity trackers have the potential to be integrated into physical activity interventions, yet little is known about how adolescents use these devices or perceive their acceptability.

**Objective:**

The aim of this study was to examine the usability and acceptability of a wearable activity tracker among adolescents. A secondary aim was to determine adolescents’ awareness and use of the different functions and features in the wearable activity tracker and accompanying app.

**Methods:**

Sixty adolescents (aged 13-14 years) in year 8 from 3 secondary schools in Melbourne, Australia, were provided with a wrist-worn Fitbit Flex and accompanying app, and were asked to use it for 6 weeks. Demographic data (age, sex) were collected via a Web-based survey completed during week 1 of the study. At the conclusion of the 6-week period, all adolescents participated in focus groups that explored their perceptions of the usability and acceptability of the Fitbit Flex, accompanying app, and Web-based Fitbit profile. Qualitative data were analyzed using pen profiles, which were constructed from verbatim transcripts.

**Results:**

Adolescents typically found the Fitbit Flex easy to use for activity tracking, though greater difficulties were reported for monitoring sleep. The Fitbit Flex was perceived to be useful for tracking daily activities, and adolescents used a range of features and functions available through the device and the app. Barriers to use included the comfort and design of the Fitbit Flex, a lack of specific feedback about activity levels, and the inability to wear the wearable activity tracker for water-based sports.

**Conclusions:**

Adolescents reported that the Fitbit Flex was easy to use and that it was a useful tool for tracking daily activities. A number of functions and features were used, including the device’s visual display to track and self-monitor activity, goal-setting in the accompanying app, and undertaking challenges against friends. However, several barriers to use were identified, which may impact on sustained use over time. Overall, wearable activity trackers have the potential to be integrated into physical activity interventions targeted at adolescents, but both the functionality and wearability of the monitor should be considered.

## Introduction

In recent years, there has been a proliferation of commercially available wearable activity trackers (eg, Fitbit, Misfit, Garmin, Apple Watch) on the market. The popularity and appeal of these devices, combined with decreasing costs, have resulted in a significant uptake by individuals to self-monitor physical activity levels, such as how many steps they take [1,2]. However, empirical research supporting the use and benefits of such devices is still emerging. To date, researchers have tended to focus on the validity and/or reliability of such wearable devices for measuring a range of outcomes in laboratory and free-living settings, including steps, distance traveled, energy expenditure, and sleep [3,4]. Research conducted with adults suggests that wearable devices have good validity for measuring steps in both settings, but lower validity for active minutes and generally poor validity for sleep outcomes [5-7]. Although comparatively little research has been conducted among youth, similar validity findings have been reported [8,9].

More recently, researchers have begun integrating wearable devices into physical activity promotion interventions in a range of populations [10-13] and tracking patients’ habitual activity and/or sleep over longer periods of time [14]. Most of the research has been conducted in adult populations, with few using these devices in interventions targeted at youth [15]. Fundamental to these interventions is an expectation that individuals know how to use the technology [16] in order to engage with and sustain their use of the device and accompanying app over a period of time (eg, weeks, months [10,11]). In the context of interventions, studies typically report an individual’s engagement with the device over time (eg, how many days of data were recorded [11,17]), and examine whether the wearable activity tracker had an impact on behavioral outcomes [15,18]. However, little research has focused on the acceptability of using these devices, particularly among youth who are active users of a range of digital devices and have had greater exposure to technology from a younger age [15,19]. Moreover, few have examined how the individual perceived and used the wearable activity tracker [20]. This is important to establish in adolescents, who are unlikely to be motivated by long-term health concerns as compared with adults [21], and perceptions of such technology may differ.

One of the biggest concerns associated with wearable activity trackers is whether individuals continue to engage with the technology over longer periods of time [22]. For example, Hermsen and colleagues found across their study that 2% of participants per week stopped using the wearable tracker entirely after being provided with the device, and 50% no longer used the technology after approximately 6 months [23]. Interestingly, increasing age was related to sustained use [23]. In contrast, despite their high use of technology generally, some studies conducted with adolescents suggest that wearable activity tracker usage reduces after approximately 2 weeks [19,24]. There is clearly a need to further examine perceptions and engagement of youth with wearable devices. Such information would provide critical insights into potential facilitators and barriers to ongoing use [24], which, in turn, has the potential to inform the development of future interventions and integration of these technologies into broader health promotion programs.

An integral component of wearable activity trackers is the automation of physical activity tracking in real time [2,15]. This allows the user to self-monitor their physical activity against public health recommendations or their own goals [2,25], receive feedback via a visual display (device and/or an accompanying app), and receive prompts or cues to be active (eg, via notifications sent through the app). These are examples of behavior-change techniques that are known to change behavior [26]. Notably, several reviews have found that up to 30 well-established behavior change techniques are present across a number of wearable devices (eg, Fitbit Flex, Garmin Vivofit, Jawbone UP, Polar Loop [2,27]) and their range of different features or functions. Such features include social support and social comparison, which may motivate adolescents to be active, given that peer influence is associated with health behaviors such as physical activity [28,29]. However, little research has examined users’ awareness and use of the different features or functions of such devices or apps with youth [1,30]. Ascertaining how to change behavior through targeting specific aspects of the device and/or app, for example, would help to inform the development of future interventions.

Therefore, the aim of this study was to examine the usability and acceptability of a wearable activity tracker among adolescents. A secondary aim was to determine adolescents’ awareness and use of the different functions and features incorporated into the wearable activity tracker and accompanying app.

## Methods

### Overview

This study drew on data collected via focus group discussions conducted with adolescents aged 13-14 years after they were given a Fitbit for a 6-week period. Participants had not previously owned or used a Fitbit. The project received ethics approval from Deakin University Human Ethics Advisory Group (Health) and the Victorian Department of Education and Training.

### Participants and Settings

Secondary schools located within an approximate 40 km radius of Deakin University Burwood Campus were identified using the publicly available My Schools website and stratified into tertiles of area-level socioeconomic status (SES) using the Socio-Economic Index for Areas [31]. A stepwise approach to recruitment was undertaken. Specifically, within each tertile, a random number generator identified the order in which schools were invited to participate in the study. In the event that the first school contacted declined the invitation, the next school on this list was contacted. This approach was continued until one school in each tertile agreed to participate in the study. Three schools (one low, one medium, and one high SES) returned informed written Principal consent (total response rate: 38%, 3/8).

Adolescents in year 8 (aged 13-14 years) were randomly selected by a school liaison teacher and invited to participate in the project. The research team was not involved in the participant selection process. A minimum age of 13 was used due to the terms and conditions stipulated by Fitbit concerning the age of use. As this was a formative evaluation study, we aimed to recruit approximately 20 adolescents (10 boys, 10 girls) per school. To be eligible to participate in the project, students could not currently own or have previously used a Fitbit monitor. Sixty adolescents from 3 schools were invited, and all provided written parental consent and student assent to take part (60/60 or 100% response rate). The participants were evenly split across school and sex.

### Wearable Activity Tracker

This study investigated the acceptability and usability of the Fitbit Flex (San Francisco, California, United States). Acceptability was defined as the perceived usefulness of the Fitbit Flex for tracking activity behaviors, while usability was defined as the perceived ease of use of the device. The Fitbit Flex is a small, wrist-worn monitor that collects minute-by-minute real-time information on steps taken, estimated energy expenditure, physical activity intensities, and sleep. Twenty behavior change techniques are integrated into the Fitbit and accompanying app [2]. Feedback is provided to the wearer through a visual display that consists of 5 light emitting diodes (LEDs) that light up as the user progresses toward their preset daily goal (1 light equates to 20% of daily goal). The Fitbit Flex wirelessly syncs data to a Web-based account (typically through a smartphone or Fitbit connect), which is only accessible via a personal log-in and password. The account can be created to enable the wearer to view and track their own personal statistics using the Web-based portal (free to access and download from Fitbit webpage) or the mobile phone app (free to download from the App Store or Google Play Store). The Fitbit Flex requires charging approximately every 5 days and can store data for up to 7 days without being synced to the user’s account. This device was chosen for four reasons: (1) Fitbit had the greatest market share of wearable activity trackers at the time of the study [32], (2) the relatively low cost (approximately Aus $100 dollars/device), (3) the inability to store personal data on the device or provide location details (ie, no global positioning system tracker), which was considered important for adolescent populations, and (4) the excellent reliability and acceptable validity of the Fitbit Flex in adults [5].

### Protocol

#### Six-Week Experimental Period

Each participant was provided with a Fitbit Flex (San Francisco, California, United States) and asked to wear it for 6 weeks (September 2015-November 2015). As participants had not previously owned or used a Fitbit device, the research team helped them to set up their Fitbit Flex. This setup process included creating a personal Fitbit account and familiarizing them with the basic functions and features of the device, including charging, syncing, and using the Web-based portal or mobile phone app for viewing their data. Such information is also provided within the packaging of the device. In an attempt to mirror experiences of consumers using the device following purchase, no other information was provided to the participants about the use of the monitor (eg, how frequently to wear it, how often to access their data, goal-setting).

#### Focus Group Discussions

Demographic data (age, sex) were collected via a Web-based survey completed during week 1 of the study. At the conclusion of the 6-week period, all adolescents participated in focus groups (up to 10 adolescents per group) that explored their perceptions of the acceptability and usability of the Fitbit Flex, accompanying app, and Web-based Fitbit profile. Adolescents’ awareness and use of the different functions and features were also discussed. A qualitative approach was used to respect the expert knowledge of the participants and to enable them to provide insights into their experiences [33]. The focus groups followed a semistructured format and were designed to address the adolescents’ perceptions and experiences of using the Fitbit Flex, views on the acceptability and usability of the different features and functions, potential facilitators or barriers to ongoing use, and describe their thoughts on the impact of the Fitbit (if any) on their overall activity levels. In total, 6 mixed sex focus groups were conducted (2 per school). All focus groups were conducted in a quiet area at each school by two of the authors (SKL, KR) and digitally recorded. Focus groups (mean duration 41.1 min, SD 6.7 min) were then transcribed verbatim, producing 198 pages (Times New Roman, size 12) of raw transcription data for further analysis.

### Data Analyses

Pen profiles, an increasingly utilized technique, were constructed from verbatim transcripts using a manual protocol [33,34]. Such a technique, which presents analysis outcomes via diagrams of composite key emergent themes, is considered appropriate and accessible to researchers with an affinity for both qualitative and quantitative backgrounds]. Example verbatim quotations were then extracted directly from the transcripts to further contextualize the theme. To provide an indication of the prevalence of the themes, the number of times a specific theme was mentioned across all focus group data is also presented [33].

Consistent with recommended approaches [35], one researcher (MF) independent to the project delivery team, initially read and analyzed the transcripts. These findings were then presented to another independent researcher (KAM) with expertise in qualitative analyses (eg, [34]), by means of cooperative triangulation. Having independently analyzed the transcripts, KAM then critically questioned the presented thematic analyses and challenged differing interpretations. A third independent researcher (MAM) subsequently analyzed the data in reverse from the pen profiles back to the transcripts. This process assured the reliability of the data obtained [33]. Finally, the pen profiles were presented to the lead author, who further critically challenged the data. This process allowed authors to offer alternative interpretations and interrogate the data until a consensus was reached. Overall, methodological rigor (ie, credibility and transferability) was demonstrated through verbatim transcription of data and triangular consensus procedures. Moreover, dependability was demonstrated through the comparison of pen profiles with verbatim citations and the triangular consensus processes.

## Results

### Findings

As no thematic differences between participants attending schools in high-, mid-, and low-SES areas emerged, data are presented collectively in 3 pen profiles. Two pen profiles broadly focus on facilitators and barriers to Fitbit use, whereas the third pen profile focuses on specific features and functions of the Fitbit Flex, which have been linked to particular behavior change techniques [2,26].

### Facilitators

**Figure 1:**
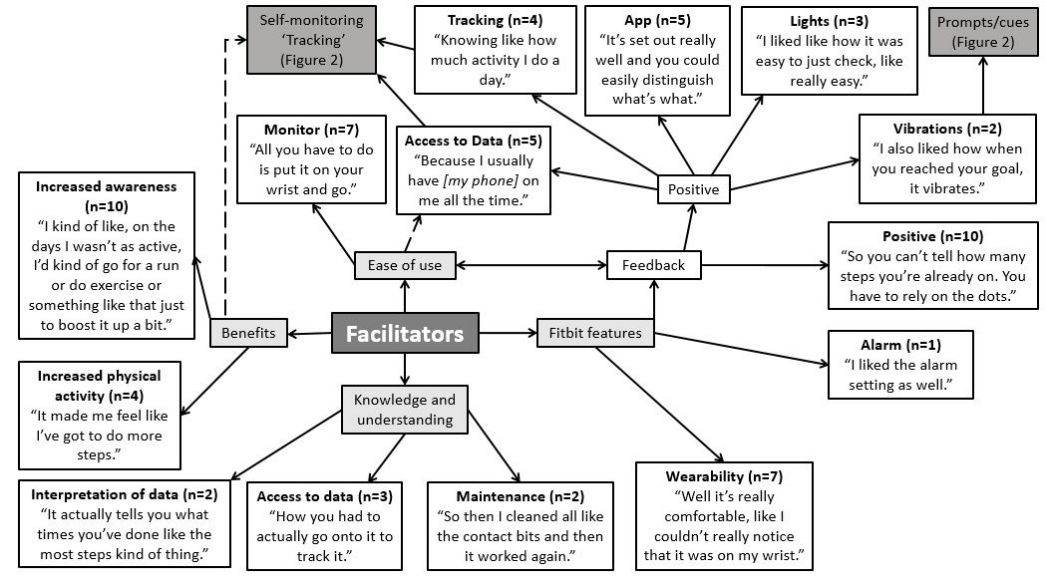
Facilitators of Fitbit use in young adolescents (n’s in brackets refer to the number of times a theme was mentioned during the focus groups). The dashed line indicates link made between different themes was noted by the researchers from the points discussed, rather than directly mentioned by the adolescents.

The facilitators of Fitbit Flex use are presented in Figure 1. Participants reported that the key enablers were the wrist-worn location, their enjoyment of tracking their physical activity levels, and ease of use as well as accessibility of data through the app. While some adolescents were content with the feedback provided through the visual display on the device (5 LED lights), others found the lack of specific feedback to be a limitation. A common theme was the desire to gain further information about their physical activity levels, such as current progress: “You can’t tell how many steps you’ve already done.” Adolescents highlighted that the ability to perform maintenance tasks (eg, clean the device, which is important for charging it), as well as the ability to easily access and interpret data through the app were key facilitators. In addition, there was a general consensus that the Fitbit facilitated awareness of and an improvement in their physical activity levels. Adolescents also felt that the vibrating function (ie, feedback that they had reached their daily step goal) reinforced positive physically active behaviors.

### Fitbit Features, Functions, and Associated Behavior Change Techniques

**Figure 2:**
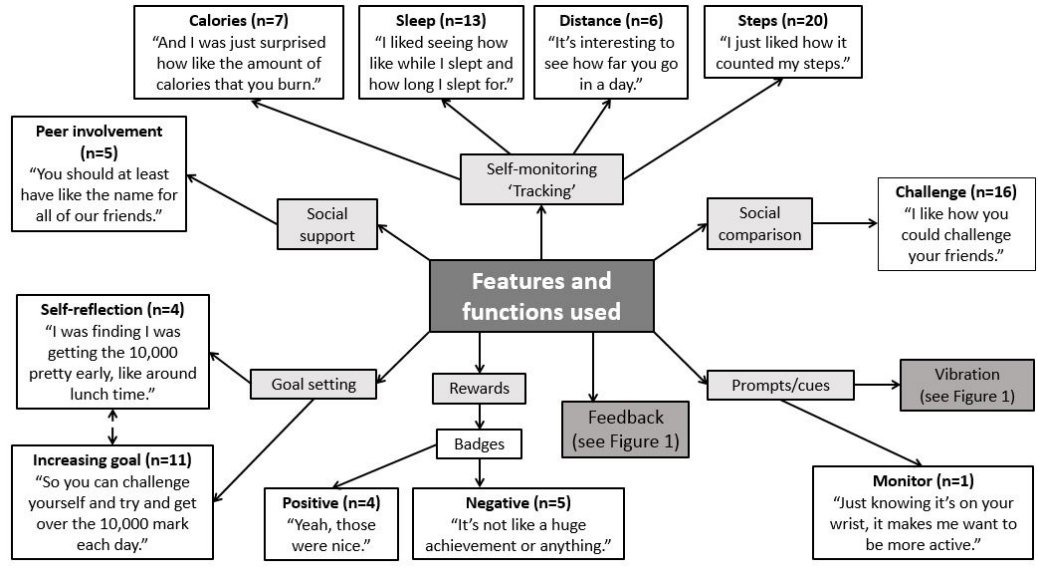
Features and functions of the Fitbit used by the adolescents.

Adolescents described a number of Fitbit features and functions that they either used or were aware of that reflected 7 specific behavior change techniques: social support, self-monitoring or tracking, social comparison, prompts or cues, feedback, rewards, and goal setting (Figure 2). Adolescents commonly reported that they enjoyed using the features that enabled them to self-monitor and obtain feedback on their steps, distance covered, calories burnt, as well as their sleep time. This was facilitated by both the visual display and the app. It was also evident that the adolescents capitalized on the opportunity afforded by the monitors to not only reflect on their daily goals but also to challenge themselves to increase them by setting new goals. Adolescents also frequently cited their enjoyment of engaging with their peers through the app, including the option to undertake challenges with their friends (eg, daily showdown). There were mixed opinions as to the motivational impact of the achievement badges (rewards) awarded via the Fitbit app for walking a set distance on a particular day (eg, 10,000 steps). Some felt motivated by these rewards to maintain their activity levels, whereas others did not feel they were a “huge achievement” and, therefore, they were not considered as an incentive as they did not value this feature. However, for one adolescent, simply wearing the monitor itself acted as cue: “Just knowing it’s on your wrist, it makes me want to be more active.”

**Figure 3:**
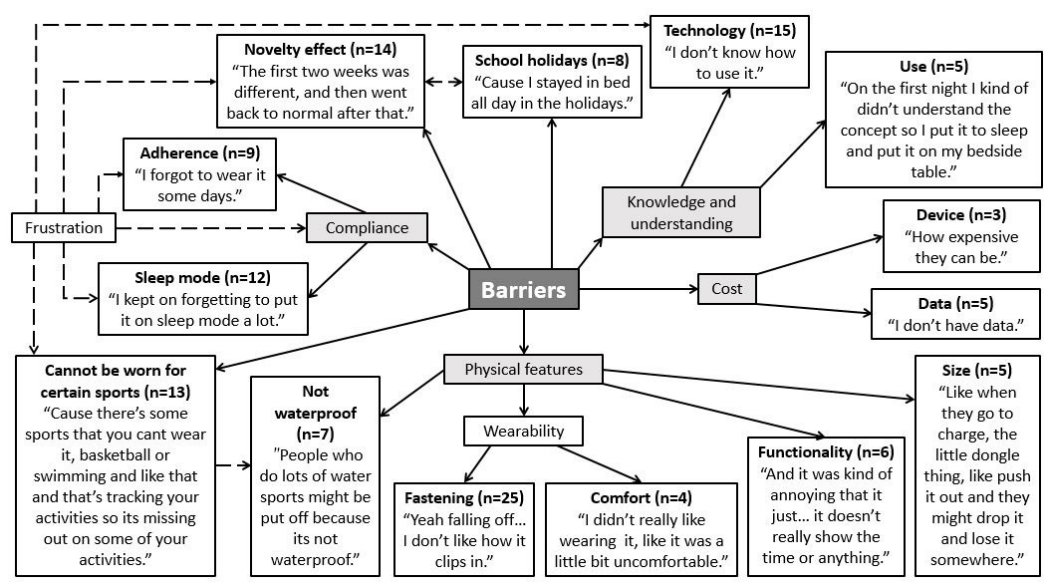
Adolescent’s barriers to Fitbit use. The dashed line indicates link made between different themes was noted by the researchers from the points discussed, rather than directly mentioned by the adolescents.

### Barriers

A number of barriers to use were raised by participants (Figure 3). These related to the design of the monitor, a lack of knowledge and understanding about how to use the device, and costs associated with using the Fitbit. Adolescents expressed frustration that the monitors were not waterproof and also could not be worn for certain sports, particularly sports such as soccer and basketball. Moreover, the fastener (ie, not secure), comfort and lack of functionality of the device per se (ie, no digital time display) were frequently cited as barriers to wearability. Furthermore, long-term compliance issues were highlighted due to problems associated with the sleep mode, which in turn was linked to frustration with the monitor (eg, not knowing how to use it), as well as a potential novelty effect after receiving the Fitbit. Adolescents reported that they had trouble either setting the monitor to record sleep or forgot to enable this feature. In addition, some adolescents identified that there was a novelty effect in terms of using the monitor to track activity, which returned back to normal after a period of time (approximately 2 weeks). However, this coincided with school holidays, which adolescents reported as a separate barrier that they thought affected their activity levels and how much they wore the Fitbit.

While Fitbits were provided to the adolescents participating in the study, in order to access their physical activity data via the app, they often had to use the data allowance that was associated with their mobile phone at a cost to the participants. This emerged as a barrier to use across all focus groups, regardless of SES. Finally, some adolescents demonstrated a lack of understanding regarding how to use various app components, such as personalizing their daily step goal (ie, increasing/decreasing goal), suggesting that participants neither used the monitor as the manufacturer intended nor accessed the various features available to them¸ particularly in the app.

## Discussion

### Principal Findings

The main aim of this study was to examine adolescents’ perceptions concerning the usability and acceptability of the Fitbit Flex when provided with the device to wear for 6 weeks. Adolescents generally found the Fitbit Flex easy to use and reported that it was a useful device for self-monitoring their day-to-day activity. They noted that this ability to self-monitor their activity increased their awareness and knowledge of their activity levels. However, a number of potential barriers were also identified, which may impact on whether they would sustain their use of the Fitbit over time. A secondary aim of the study was to examine adolescents’ awareness and use of the different functions and features incorporated into the Fitbit Flex and accompanying app. The adolescents utilized a range of functions and features of the device and app, which corresponded to 7 behavior change techniques. Together, these findings provide insights into how adolescents engaged with the Fitbit Flex, which in turn may help to identify how to integrate it into physical activity interventions for adolescents and what factors may need to be addressed to try to facilitate long-term use.

### Comparisons With Prior Work

There is currently a dearth of research that has examined the acceptability and usability of wearable devices by youth [15]. Results generally indicate that wearable devices are viewed favorably by youth, with factors such as ease of use, aesthetics, and comfort important facilitators for ongoing use [10,16,17,22,36,37]. Similar findings were observed in this study. Specifically, ease of use was an important facilitator of monitor use, with adolescents noting that the app was easy to navigate and the Fitbit Flex visual display straightforward to check. However, a number of adolescents expressed difficulties with the sleep mode in particular, which led to frustration with the device and, in some cases, the adolescents reported that this impacted on wearing the device (eg, removed overnight and forgot to put it back on the following day). This suggests that the user may need assistance with using features or functions of wearable devices. However, the assistance required may differ for different age groups. For example, previous research has indicated that adolescents may need support in personalizing the device in relation to daily goals [28]. On the other hand, research with adults and older adults indicates that training on how to use the device and the app more generally may be required [38,39]. Researchers and practitioners should consider including information as part of an intervention to ensure the device is used as intended.

There were contrasting findings in relation to comfort and wearability, with some adolescents noting that they found it comfortable to wear on the wrist (7 mentions) while others found this was a barrier to wearing the device (4 mentions). The biggest issue noted by the adolescents was the manner in which the device fastened around the wrist (25 mentions), with concerns being raised that this was not secure. Previous research has noted that loss of a device (or fear of losing the device) is a major barrier to an individual’s experience and engagement [16]. Interestingly, a study conducted with older adults also found the Fitbit Flex locking mechanism to be problematic. This was due to fastening the clasp itself, but once closed, the older adults did not report concerns about losing the device [39]. Overall, this suggests that researchers should consider not only the function of the wearable device for use in an intervention (eg, feedback on steps, sleep) but the wearability of the device in different age groups to enhance the individual’s experience and promote compliance with the monitoring protocols.

In general, adolescents noted that they did use the Fitbit Flex to track a range of data, including steps taken, distance traveled, and sleep. This appeared to increase their awareness of their activity levels, with several adolescents noting that they used this feedback to increase their overall activity levels. Others have noted similar findings, suggesting that the use of a wearable device can trigger short-term increases in physical activity levels [24], particularly when the step target had not been achieved [19]. This is a positive finding in the context of using the devices within an intervention, as without knowledge of their current activity levels, youth are unlikely to change their behaviors as they may see no need to do so [40]. It is important to note that feedback is also an important component for increasing awareness [40], yet several issues were raised relating to the feedback provided by the Fitbit Flex. Some adolescents reported that the visual display on the device provided sufficient feedback against their day goal, yet others found that this was inadequate and, in some cases, this was identified as a barrier to use. In particular, adolescents expressed interest in knowing their actual steps rather than relying on the lights for information. This might be explained, in part, by the current adolescents participating in a step challenge within the app to compete against their peers, for which knowing how your daily step score compared with others may have been critical to success (or not). While detailed step feedback is available through the Fitbit app, some adolescents noted that accessing this information came at a cost as they had to use their mobile phone data allowance. As previous research has found that youth tend to prioritize use of their data for entertainment purposes [41], it is possible that these adolescents did not access the app frequently [if at all] to view their data and did not receive specific feedback on their daily steps. Overall, researchers may need to consider using devices that provide specific feedback on the display (eg, steps), particularly in this population, to address these issues.

A positive finding was that adolescents reported using a range of features and functions of the Fitbit and the accompanying app, which are based on behavior change techniques that are known to influence behavior [22,26]. In addition to self-monitoring, goal setting, and feedback, peer involvement (either as support or comparisons) was frequently cited as an element of the app (in particular) that the adolescents liked and used. This supports previous research that noted that competition with peers and peer-surveillance promoted social connections [19]. Future interventions using wearable devices should consider how to capitalize on these features that promote peer involvement (eg, daily challenges), particularly given the influence of peers on adolescents’ physical activity [42]. However, this must be balanced with promoting self-comparisons and autonomy and ensuring that activity engagement is self-determined. Indeed, some have suggested that competition can increase negative feelings of self, and adolescents have reported engaging in activity due to peer pressure [19]. As a consequence, adolescents may remove themselves from engaging in peer-surveillance (eg, sharing activity levels with others) altogether [19,28].

Interestingly, other features of the app that are considered to reward physical activity behavior (eg, step badges) were not particularly valued by most and, in general, were not seen as a significant achievement. This is in contrast to previous studies in adolescents that combined a Fitbit Flex with a Facebook group, where the badges were seen as a reward for effort, reinforced activity behaviors, and also provided opportunities for social comparison and support within the group [12,37]. Those results may be due to adolescents being able to see other group members’ achievements, which introduced an element of social comparison, competition, or support (ie, achieving badges as a group; [12]). It is possible that factors such as motivation regulation or stages of change may moderate the acceptability and usability of specific features of the Fitbit [24,28]. As an example, the Fitbit app awards badges to reward individuals, and those with higher autonomous motivation or readiness to change their physical activity behavior may perceive these badges differently to those with lower autonomous motivation. Examining how individuals perceive such features that are designed to reward daily and sustained effort (eg, distance badges) may be warranted to identify how (if at all) they can be utilized within wider programs using wearable technology. Overall, the findings of this study provide insights into potential features embedded into the device and/or app, which are based on behavior change techniques that could be targeted within an intervention to increase activity levels. However, it is important to note that these are likely to be device-specific and may not apply to other wearable activity trackers, and the usability of the linked features may dictate whether or not these features should form a specific component of an intervention [2].

One of the concerns of wearable technology is whether an individual sustains their use of the device over the longer term [22]. It has been reported that use of wearable devices declines over time, with approximately 25% to 50% of adults ceasing to use the technology within the first 6 months of ownership [22,23]. This is consistent with previous studies that have utilized self-monitoring of behaviors [43]. Despite the adolescents in this study using the Fitbit Flex for 6 weeks, it was noted by participants that there was a novelty effect of using the device, which started to wear off after the first couple of weeks of use. This finding supports previous studies conducted with adolescents, where interest waned after 2 to 4 weeks of use [19,24,28]. This may suggest that the devices could be a useful first step in helping to establish an individual’s awareness of activity levels but other techniques may need to be targeted (eg, via the app or additional resources) to facilitate sustained use over longer periods of time. Alternatively, this may indicate that during this initial period when motivation and interest are likely to be high [24], researchers can capitalize on this window of opportunity within an intervention to try to integrate the device into a feature of daily living. Addressing factors that lead to sources of frustration, such as knowledge of the technology, how to use the device, and how to access and interpret data may be critical during this time to try to encourage the wearer to continue to use the technology. Previous studies have reported helping participants to know how to use the device and understand the collected data when the device is distributed [10,11,38], though additional information may need to be provided during an intervention to manage issues such as expectation mismatch (eg, technology is not doing what the user expected), which are a common reason for ceasing to use wearable devices [44].

The strengths of this study include the assessment of the feasibility of a wrist-worn wearable device in young adolescents in free-living settings, the inclusion of adolescents from low-, medium-, and high- SES backgrounds, and the use of qualitative methods to explore adolescents’ thoughts and experiences in depth. However, there are several limitations that should also be noted. First, the liaison teacher at participating schools was asked to randomly select adolescents to receive an invitation to participate. The only selection criterion that we stipulated was no previous use or ownership of a Fitbit device. However, as we were not involved in the random selection of students, it is possible that students may have been specifically invited based on characteristics unknown to the research team to participate by their teacher. Second, the Fitbit used during the study has since been superseded by a newer model, which has addressed issues relating to waterproofness and automatic sleep tracking. These were 2 barriers identified by the adolescents in this study. Third this study assessed the adolescents’ experiences after a 6-week period. While this study provides insights into their initial experiences of using the device, it is not known how this may have influenced long-term use, if at all. Longer-term studies are needed to establish how use of the device changes over time. Finally, no information was collected from the adolescents concerning their experience of using other wearable devices. It is possible that they had previously used other devices (eg, Garmin, Jawbone), which may have influenced their perceptions of the Fitbit Flex. However, it should be acknowledged that none of the adolescents in the focus groups compared their experiences of the Fitbit Flex with other wearable devices that were commercially available at the time of the study.

### Conclusions

Wearable devices provide an opportunity to promote physical activity to adolescents, yet little is known about how youth engage with and use such technology. Overall, this study found that a wearable device (Fitbit Flex) was highly acceptable to adolescents, and the device was used to self-monitor activity levels (and other behaviors). Adolescents reported using a range of functions and features that could be integrated into comprehensive physical activity interventions, reinforcing the potential of these technologies for promoting activity levels in this population. Potential issues were also noted, which may decrease the feasibility of using such technology within an intervention or health promotion program, though barriers related to knowledge of using the different functions and interpreting data can be addressed using supporting techniques. Other barriers related to the specific device used, highlighting that these must also be considered during intervention development.

## Abbreviations

LED: light emitting diode
NHMRC: National Health and Medical Research Council
SES: socioeconomic status
